# Method of surface energy investigation by lateral AFM: application to control growth mechanism of nanostructured NiFe films

**DOI:** 10.1038/s41598-020-71416-w

**Published:** 2020-09-01

**Authors:** T. I. Zubar, V. M. Fedosyuk, S. V. Trukhanov, D. I. Tishkevich, D. Michels, D. Lyakhov, A. V. Trukhanov

**Affiliations:** 1 SSPA “Scientific and Practical Materials Research Centre of NAS of Belarus”, P. Brovki str., 19, 220072 Minsk, Belarus; 2 South Ural State University, Lenin Prospect, 76, Chelyabinsk, Russia 454080; 3 Computer, Electrical and Mathematical Science and Engineering Division, King Abdullah University of Science and Technology, Thuwal, 23955-6900 Saudi Arabia; 4 National University of Science and Technology MISiS, Leninsky Prospekt, 4, Moscow, Russia 119049

**Keywords:** Nanoparticles, Two-dimensional materials, Atomic force microscopy

## Abstract

A new method for the specific surface energy investigation based on a combination of the force spectroscopy and the method of nanofriction study using atomic force microscopy was proposed. It was shown that air humidity does not affect the results of investigation by the proposed method as opposed to the previously used methods. Therefore, the method has high accuracy and repeatability in air without use of climate chambers and liquid cells. The proposed method has a high local resolution and is suitable for investigation of the specific surface energy of individual nanograins or fixed nanoparticles. The achievements described in the paper demonstrate one of the method capabilities, which is to control the growth mechanism of thin magnetic films. The conditions for the transition of the growth mechanism of thin Ni_80_Fe_20_ films from island to layer-by-layer obtained via electrolyte deposition have been determined using the proposed method and the purpose made probes with Ni coating.

## Introduction

The permalloy or Ni–Fe alloy (45–82% Ni) is widely used material due to a unique combination of magnetic and functional properties^1–5^. Permalloy with the chemical composition of ~ Ni_80_Fe_20_ is most often used for practical applications, because it has excellent magnetic softness, high permeability, low coercivity, small magnetic anisotropy and almost zero magnetostriction^6,7^. The most common applications of the Ni_80_Fe_20_ are the manufacture of electromagnetic shields for the protection of the functional electronics^8,9^ and sensitive elements of magnetic field sensors based on anisotropic and giant magnetoresistance effects^10,11^. A large interest in thin Ni_80_Fe_20_ films and partially filled granular 2d-structures is manifested from a fundamental point of view due to the possibility of the formation of anomalies magnetic phenomena like skyrmions and vortex–antivortex pairs^12–14^.

A lot of research is being done to get the materials with the desired functional properties^15–17^. In most cases, the microstructure determines the properties^18,19^, especially in the case of nanostructured materials, thin films, multilayer structures, etc. For this reason, a simple way to create materials with the necessary properties is to control the microstructure at the synthesis stage. Many attempts have been made to control the film growth mechanisms, and authors agree that the implementation of one or another growth mechanism depends on the ratio of the interaction or binding energy between the substrate and the nanograins (NGs) of the synthesized material. For example, when the binding energy between deposited atoms and the substrate surface (E_IA-S_) is less than the binding energy between initial and film atoms E_IA-FA_, film deposition is similar to deposition onto a non-wetting surface and Volmer–Weber (island) mechanism of film growth is carried out. Otherwise, when E_IA-S_ > E_IA-F_, the deposition occurs by two-dimensional layer by layer growth mechanism^20^. Thus, the controlling the binding energy makes it possible to change the growth mechanism and, therefore, to manage a film microstructure and properties of synthesized material^21,22^.

There is no acceptable technique for estimating surface energy in a microscopic local area or on a separate NGs at present. The universal and accurate method of surface energy investigations is based on the assessment of the wettability of a surface^23,24^, but this allows us to estimate the total surface energy of the sample, but not a separate microscopic area or NG. The best contender for determining local specific surface energy is an atomic force microscope (AFM). There is a technique for determining local surface energy with AFM, which is called force spectroscopy^25,26^. It is based on measuring the bend magnitude of the probe cantilever when it is snap-off the investigated surface (perpendicular or normal movement). The magnitude is proportional to the force required to separate the probe and the surface. The value of the normal specific surface energy (SSE) can be obtained using contact interaction models (Bradley^27^, Johnson–Kendall–Roberts^28^, Derjagin–Muller–Toporov^29^, Hertzian^30^, etc., depending on contact conditions). This technique is widely used to study biological objects, less often for polymers, but is poorly applicable for the studying of materials with low adhesion, such as metals and alloys. In addition, there is a limitation of the force spectroscopy method when conducting research in the atmosphere of air. The water layer adsorbed on the surface has a significant effect on the investigation results and leads to measurement errors. It is recommended that the surface energy be determined only in a liquid cell^31–33^ or in climate chambers. Most authors agree that the investigations of surface energy under atmospheric conditions using AFM is qualitative and comparative results, which can be used for mapping of the material phases with various properties. For example, the adhesion properties between the AFM tip and the sample surface in liquids such as water, alcohol, formamide, and an aqueous solution of potassium chloride were measured in Ref.^33^.

Another method for studying surface properties using AFM is based on measuring the cantilever during surface scanning in contact mode. The twist of the cantilever is proportional to the frictional force between the tip and the investigated material. This method makes it possible to assess nanoscale tribological characteristics (friction and wear) and is unique today^34–41^. Despite the fact that there is no alternative method for studying nanoscale friction, the method of atomic force tribology has not yet been sufficiently studied. It is known that completely different processes begin to influence the friction with the transition to the nanoscale. The forces of van der Waals interaction, the formation and destruction of chemical bonds, electromagnetic interaction, adsorption processes, and much more make a significant contribution to nanofriction and is negligible at the macrolevel friction^42–48^. Therefore, it is impossible to extrapolate friction mechanisms from macro- to micro- and nanoscales. For example, on the nanoscale (contrary to classical macrofriction) there is no direct relationship between force and coefficient of friction and normal load^49–52^. In addition, many authors have shown that nanofriction is dependent on the scanning rate and attributed this to the rate of bond formation in the contact of the tribopair^48,53,54^. The authors of some works^35,36,38,40,42,43,55^ indirectly relate the friction force investigated using AFM with the surface energy of the material, since both of these forces are of a similar nature.

In the paper, we propose a new technique for specific surface energy investigations suitable for studying separated NGs or microscopic local area. Based on the classical models of contact interaction and the widely known method of studying nanofriction using AFM^56,57^, we have developed a new approach to predicting the behavior of NGs. This made it possible to explain the anomalous transition of the growth mechanisms of thin Ni_80_Fe_20_ films. There was a transition from qualitative conclusions based on indirect factors to a quantitative assessment of the causes of changes in NGs behavior. This not only deepens the understanding of the behavior of nanograins in the process of film formation, but also takes a step towards controlling this behavior.

## Results and discussion

### Development of the method for specific surface energy investigation

**Figure 1:**
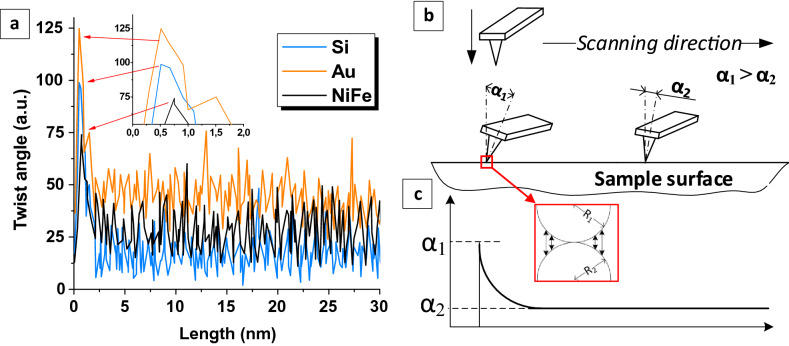
Cantilever twist recording during the AFM-scanning Si (100) surface, thin Au film and nanostructured Ni_80_Fe_20_ film obtained in SP-mode (**a**) and schematic representation of the AFM-probe behavior during scanning (**b**) with an idealized graph of the twist angle (**c**).

A new AFM method has been developed for determining surface energy in local area such as individual NGs or other surface part of interest. The local separation method is based on the method of nanofriction investigations^58,59^. Nanofriction can be investigated by recording the AFM probe twisting during surface scanning in the contact mode. The friction force obstructs the probe movement and cantilever twists. The result of nanofriction investigation using AFM is torsion profile (probe twist angle vs. scanning length). The torsion profiles of the Si surface with crystallographic orientation (100), thin (~ 10 nm) Au film and nanostructured Ni_80_Fe_20_ film obtained in SP electrodeposition mode are shown in the Fig. 1a. After analyzing a large number of torsion profiles, it was noticed that the initial section contains a peak value of twist or a region of the cantilever twists extremely tightly. This peak is not more than 1 nm from the first point of movement (see the enlarged insert in the Fig. 1a. Figure 1b shows the behavior of the probe in the initial stages of AFM scanning. The result of the twist recording is presented in idealized form in the Fig. 1c. First, the probe approaches the surface perpendicular to it. Then, the movement of the table with the sample begins and the cantilever is twisted until the force allows to separate the surface and the tip. As the result, the twist angle *α*_1_ reaches the maximum at the moment just before the start of the sliding motion. This maximum twist angle (*α*_1_) corresponds to the peaks in Fig. 1a. After that, the probe moves along the surface and experiences the opposition of the sliding friction force, that is why it twists at a relatively constant angle *α*_2_ (*α*_1_ > *α*_2_).

In macroscale mechanics, there is a concept of the rest or static friction force (*F*_*st*_) that impedes the motion beginning, which is always greater than the sliding friction force (*F*_*fr*_), *F*_*st*_ > *F*_*fr*_. However, the nanoscale interaction has a completely different nature. The main part of the interaction force is the Van der Waals force, electrostatic and magnetic interaction, sometimes the formation of chemical bonds, etc. Thus, the analysis of the torsion profiles makes it possible to study the behavior of nanosized materials in contact and value of their interaction.

It is known that SSE is the energy necessary to create a surface unit. Obviously, this occurs when a probe transitions from a static state. Therefore, to determine the surface energy *γ*, it is necessary to determine the force that counteracts the onset of motion *F*_*st*_, and then subtract the sliding friction force *F*_*fr*_ from this value. So we can get the lateral separation force *F*_*ls*_, which is proportional to the energy. In general, the lateral separation force *F*_*ls*_ is calculated in accordance with the following equation

1 $$F_{ls} = \, F_{st} - \, F_{fr}.$$

In the present work, we adapt the contact mechanics models used to study adhesion via AFM force spectroscopy for the numerical determination of SSE (*γ*). The Bradley model is used to describe the interaction mechanism of two rigid spheres. The assumption is made in the work that the AFM tip has a spherical shape that is constant during scanning. NiFe NGs are also close to spheres, which is confirmed by their morphology, studied even in the article^59^. Thus, this model is suitable for contact between the AFM tip and NGs or surface with roughness *Ra* > 0. In accordance with the Bradley model, a force

2 $$F = - 2\pi \gamma R$$

must be applied to separate the surfaces.

In Eq. (2), 1/R = 1/R_1_ + /R_2_ (R_1_ and R_2_ are radii of two interacting spheres), so R is) a radius of the contact area. The following equation for SSE calculation (2) is the result of adapting contact interaction models to the described case of contact with AFM probe.

3 $$\gamma = - \frac{lk}{{6\pi s\left( {1 + \vartheta } \right)}} \cdot \left[ {\int\limits_{0}^{L} {\left( {f_{fw} \left( x \right) - F_{fr} } \right)} dx + \int\limits_{L}^{0} {\left( {f_{bw} \left( x \right) - F_{fr} } \right)} dx} \right]$$

where *l* is a cantilever length, *k* is a cantilever stiffness coefficient, *a* is a tip radius, *s* is a tip height, *υ* is a Poisson's ratio of NiFe NGs and silicon, *L* is a length of scanning line, *f*_*fw*_ and *f*_*bw*_ is a function of the average torsion profiles obtained in the forward and reverse motion, *F*_*fr*_ is a sliding friction force, which can be calculated as

4 $$F_{fr } = \frac{{lk(z_{fw} - z_{bw} )}}{{6s\left( {1 + \vartheta } \right)}}$$

where *z*_*fw*_ and *z*_*bw*_ are the average cantilever deviations in the forward and reverse motion, respectively. A detailed description of the processing of profiles is in the article^60^.

### SSE investigation method testing

**Figure 2:**
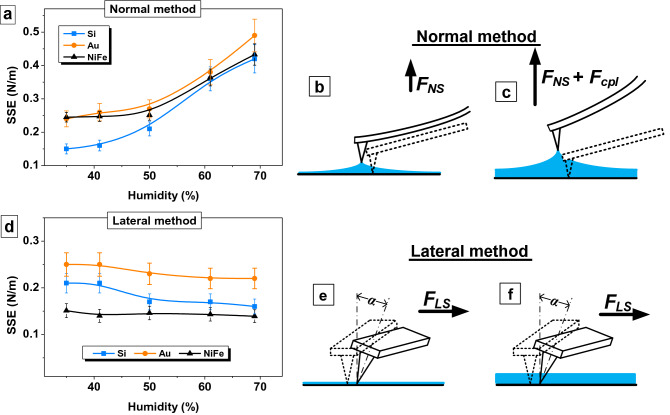
Testing developed SSE determination method and comparing it with the method of AFM force spectroscopy. (**a**) Influence of air humidity on SSE measurement results obtained with force spectroscopy (normal separation method); (**b**) influence of air humidity on SSE measurement results obtained with lateral separation method; (**c**,**d**) a scheme of the influence of moisture on the probe behavior for the lateral method; (**e**, **f**) for the lateral method.

Figure 2 shows the influence of air humidity on the results of determining the SSE of single-crystal silicon with crystallography orientation (100), thin golden film and nanostructured permalloy film obtained in SP mode. The force spectroscopy method or normal separation method (Fig. 2a–c) and the lateral separation method developed in this work (Fig. 2d–f) were used for testing the method that we proposed. The result shows that SSE determined using the normal method increases several times with an increase in humidity from 34 to 69% (Fig. 2a). So, the SSE of the silicon was 0.15 N/m at 35% humidity and 0.42 N/m at 69% air humidity. There is almost 3 times increase. Similar results were obtained for all samples studied using the AFM force spectroscopy method. The SSE of gold increased from 0.24 to 0.49 N/m, and from 0.24 to 0.43 N/m for Ni_80_Fe_20_ film. All these results may not be correct, because surface energy cannot increase several times due to humidity. The error in the measurements by the normal separation method appears due to the presence of an adsorbed water on the surface. A thin layer of water forms the meniscus when the probe lift from the surface, see Fig. 2b and c. Therefore, it is necessary for the extender to overcome the forces *F*_*NS*_ + *F*_*Cpl*_ due to capillary effects to separate the probe and the surface. As a result, the AFM force spectroscopy method can be useful for investigations in vacuum, liquid cell or climate chamber but not in the air atmosphere.

The lateral separation force *F*_*LS*_ rather than normal separation force *F*_*NS*_ is used in the developed method for calculating SSE. If there is a layer of water on the surface, the tip moves inside the layer (Fig. 2e,f). Capillary forces do not affect on the investigations results. The Fig. 2b shows the values of the SSE for all investigated materials remain almost constant within the measurement accuracy over the entire humidity range. A decrease in silicon SSE from 0.21 ± 0.02 N/m to 0.17 ± 0.02 N/m can be explained by surface oxidation. Consideration should be given to the values of the Ni_80_Fe_20_ SSE. It varied from 0.24 to 0.43 N/m when measured by the normal method, and it became much lower 0.14–0.15 N/m when measured by lateral method. This can be explained by the fact that the studies were carried out by a magnetic probe. Therefore, the normal separation of the probe and surface is also hindered by the magnetic component of the interaction of the surfaces. Probably, this effect is minimal when the probe moves along the surface, as in lateral method. So, the testing proved the suitability and good accuracy of the method for evaluating the energy of various materials in a wide range of humidity.

### Application of the SSE investigation method to control the growth mechanism of Ni_80_Fe_20_ films

The chemical composition of NiFe films was studied by energy-dispersive X-ray microanalysis (EDX). The Ni/Fe ratio in the synthesized film was 80/20 at% ± 3%. This suggests that the chemical composition depends on the electrodeposition technological regime insignificantly. The closest Ni/Fe ratio to the desired composition was obtained in DC mode, it was Ni_80.38_Fe_19.62_ (Table 1). A slight deviation from stoichiometry was observed after the transition to pulsed modes. The sample obtained in LP mode contained 78.36 at% nickel and 21.64 at% iron and the sample deposited in MP-mode had 76.95 at% nickel and 23.05 at% iron. There was a slight (about 3%) increase in the iron content. A chemical composition gradient in NiFe thin films near the substrate with subsequent stabilization of the composition for thick coatings is a known phenomenon found in binary (Ni–Fe, Co–Cr, Co–Fe) and ternary (Co–Ni–Fe) alloys obtained by electrodeposition^61,62^. The composition gradient is usually an increase in the iron content near the substrate that was observed for the samples obtained in LP and MP-modes. However, the film obtained in SP mode had almost 3% decrease in iron compared with the DC sample. Earlier^63^, we observed this phenomenon and it was associated with the change in the growth mechanism.

**Table 1:** The nominal chemical composition of the Ni_80_Fe_20_ films.

	DC-mode	LP-mode	MP-mode	SP-mode
Ni content (at%)	80.38	78.36	76.95	82.89
Fe content (at%)	19.62	21.64	23.05	17.11

**Figure 3:**
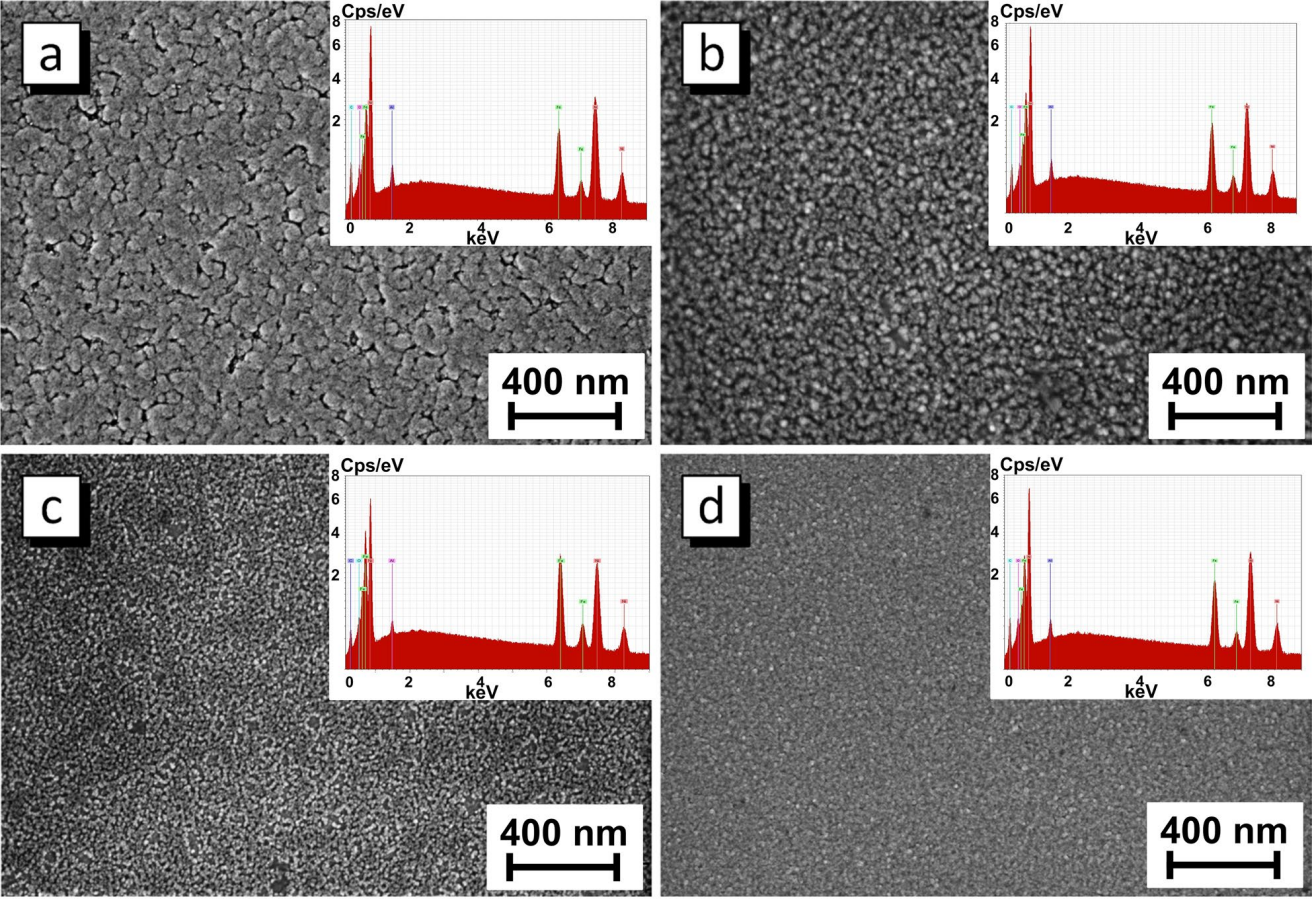
Surface microstructure and EDX-spectra of the Ni_80_Fe_20_ films obtained in DC (**a**), LP (**b**), MP (**c**) and SP modes (**d**).

**Figure 4:**
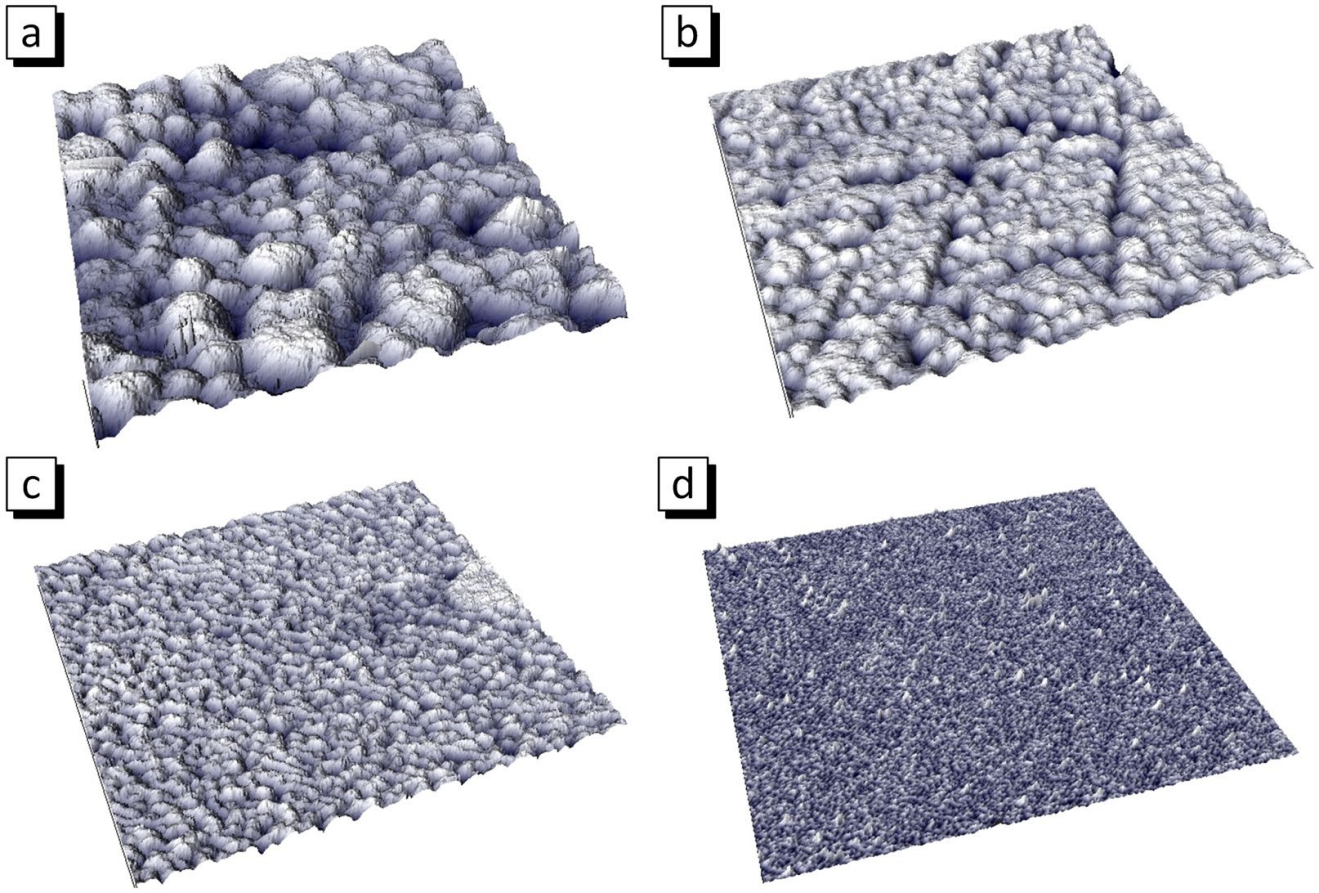
AFM morphology of Ni_80_Fe_20_ thin films obtained in DC (**a**), LP (**b**), MP (**c**) and SP (**d**) modes, size of the images is 1 µm^2^.

Figures 3 and 4 show the surface microstructure evolution of the sample obtained in different electrodeposition modes. The average thickness of film obtained at direct current is about 800 nm (Table 2). The stationary electrodeposition mode with direct current provides the synthesis of films with a pronounced grain structure. Analysis of AFM 3d image (Fig. 4a) allows us to conclude that grains contains a large number of NGs, that is the grains is a NGs agglomerates.

**Table 2:** Thickness, average NGs size and roughness of Ni_80_Fe_20_ films obtained in different electrodeposition modes.

	DC-mode	LP-mode	MP-mode	SP-mode
Average thickness (nm)	800	350	80	10
Average NGs size (nm)	45	25	12	≤ 5
Roughness (nm)	14.7	11.3	6.6	1.5

The films obtained in LP and MP modes has a similar structure. There are agglomerates including NGs with an average size of about 25 nm for LP mode and 12 nm for MP mode. The thickness of the LP mode film is about 350 nm. The reason for 2.2 times thinning compared with the DC film is that the effective deposition time is 2 times less in the pulsed mode (15 s). The thickness of the film obtained in MP mode is 80 nm. This is 4.4 times less than the film with equal effective deposition time. This can be explained by the imperfect leading edge of a pulse. If the edge is not perfectly vertical, then the current will be lower than the set value a while until the desired current value is set. This is repeated for every pulse. The larger the number of pulses, the longer the deposition takes place at low current. This is probably why the film thickness decreases as the number of pulses increases. So, there are 30 pulses for 30 s deposition in LP mode, 3 × 10^4^ pulses in MP mode and 3 × 10^6^ pulses in SP mode. As a result, the Ni_80_Fe_20_ film with average thickness about 350 nm was deposited during 30 s in LP mode, 80 nm in MP mode and 10 nm in SP mode.

The structure of Ni_80_Fe_20_ film obtained in SP mode is different. The NGs have the size of about 5 nm, do not combine into agglomerates, form a uniform layer with surface roughness of 1.5 nm. The information about NGs size and surface roughness is given in Table 2. The decrease of the NGs size occurs because a new crystalline is formed with each pulse and grows until the pause. The transition from direct current to pulsed electrodeposition modes and a decrease in pulse duration made it possible to significantly reduce the NGs size from 45 nm for DC film to 5 nm for film with the shortest pulses (SP mode). This may be useful for the synthesis of the magnetic field sensors materials operating on GMR and AMR effects. The surface roughness decreased from 14.7 nm for DC film to 1.5 nm for the film obtained in SP mode due to the reduced NGs size as well as due to the lack of agglomerates and uniform film formation. Thus, the microstructure shows that the films grow according to the island mechanism in DC, LP and MP modes and in layers in SP electrodeposition mode.

**Figure 5:**
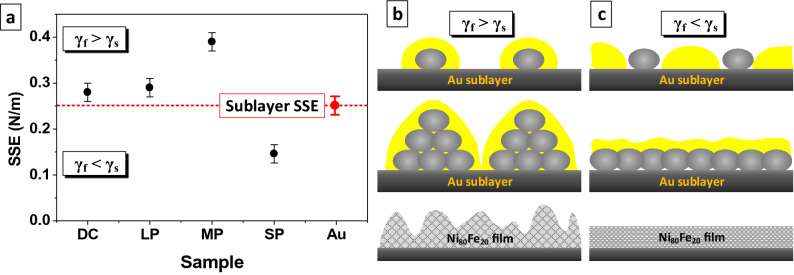
Values of SSE of the Ni_80_Fe_20_ films and Au sublayer SSE measured using lateral separation method (**a**) and the mechanisms of the Ni_80_Fe_20_ films growth for DC, LP and MP modes, when *γ*_*s*_ > *γ*_*f*_, by island mechanism (**b**) and for SP mode, when E_IA-SS_ > E_IA-FA_, by layered mechanism (**c**). The yellow areas are a more probably place to nucleation of new grains.

Figure 5a demonstrates the values of SSE of Ni_80_Fe_20_ films obtained in different electrodeposition modes (black circle) and Au film that was used as the sublayer for magnetic films deposition (red circle). The measurements were carried out using the lateral separation method on a nanoscale site in the center of the NG. The SSE values nonlinearly decreased during the transition from DC deposition mode to pulsed modes and decrease in pulse duration. This trend occurs due to a decrease in NGs size. The smaller the NGs size, the larger the surface atoms that have excess free energy compared to the atoms in the NGs volume.

The results well explain the microstructure evolution of the Ni_80_Fe_20_ films shown in the Figs. 2 and 3 and the change in the growth mechanism. The SSE value of the Au sublayer surface *γ*_*s*_ was 0.25 N/m. The SSE of the NGs of the permalloy films *γ*_*f*_ obtained in the DC, LP and MP modes were 0.27, 0.29 and 0.39 N/m respectively. SSE increases because the proportion of the surface atoms increases compared to volume atoms. When the SSE of the sublayer is less than the SSE of the film NGs (*γ*_*f*_ > *γ*_*s*_ ) the most probably position for the formation of a new NGs is on the existing NGs. The NGs form conglomerates and the film grows according to the island mechanism^64^. The film growth schematic illustration in such conditions is shown in the Fig. 5b. The most probably positions highlighted in yellow in the schematic illustration. The island growth mechanism is being implemented in DC, LP and MP electrodeposition modes, and as a result, the film with a high roughness and thickness heterogeneity is obtained. The value of SSE of Ni_80_Fe_20_ films obtained in SP mode was 0.15 N/m. An ultra small grain size (less than 5 nm) is formed when the pulse is shortest (10^–5^ s). The SP film is in a quasi-amorphous state, which is usually characterized by a low energy value. Ratio *γ*_*f*_ < *γ*_*s*_ is performed. In this case, the position on the substrate between the NGs is most probably, as shown in yellow in the Fig. 5c, because it has more surface energy. So, the grains fill the substrate with a continuous homogeneous layer and the film grows layer by layer in the initial stages. Such growth mechanism makes it possible to obtain a thin magnetic film with a low roughness and high uniformity of thickness.

As a result, the method for specific surface energy investigation using AFM was developed by combining the capabilities of force spectroscopy and a method for studying nanofriction. The specific surface energy was determined at the stage of separation of the tip from the surface resting point and was calculated using an adapted contact mechanics. The Bradley model of contact mechanics has been modernized in present work. The Bradley model was chosen because it describes the behavior of two rigid spherical bodies under dynamic contact. Moreover, other contact mechanics models including Hertz non-adhesive elastic model, Derjaguin–Muller–Toporov and Johnson–Kendall–Roberts models of elastic contact may also be modernized in a similar way. The model choice depends on the contact conditions. The proposed method has several advantages over previously used methods. Firstly, there is no need to use climate chambers and liquid cells to study surface energy by the AFM, because air humidity does not affect the results. This was confirmed at the stage of testing the method when comparing the SSE of silicon, gold and permalloy obtained at air humidity from 35 to 69% using the proposed method and power spectroscopy. The deviation of the SSE values did not exceed 5%. This is achieved because the direction of probe motion is changed from normal to lateral.

One of the possible applications of the method is shown in the paper. The SSE of films and individual Ni_80_Fe_20_ nanograins obtained in various modes of electrodeposition as well as gold sublayer was studied. The purpose made probes with Ni coating were used for accurate experimental modeling of the nanograins behavior in dynamic contact. It was established that the SSE of Ni_80_Fe_20_ NGs of the films obtained in the DC, LP, MP and SP modes was 0.27, 0.29, 0.39, and 0.15 N/m, respectively, and the surface energy of gold was 0.25 N/m. A strong correlation was found between the ratio of the NGs SSE and the sublayer SSE and the change in the microstructure of the films, which is caused by a transformation of the growth mechanism. It has been proven that if the film SSE is greater than the SSE of the substrate the island growth mechanism is realized. This occurs in the DC, LP and MP modes. When the substrate SSE is greater than the SSE of the deposited NGs, the film grows in layers and has a low roughness as in the SP mode in the present work. Thus, it is possible to predict and control the film growth mechanism using the method of the SSE investigation by AFM. However, the possibilities of the proposed method are not limited to controlling the mechanism of film growth and can be expanded for use in catalysis, in the study of nanostructured materials and biological objects.

## Methods

### Sample preparation

The Ni_80_Fe_20_ thin films samples were produced via electrolyte deposition in four different modes: at direct current (DC-mode) and pulsed current with pulse duration 1 s (LP-mode), 10^–3^ s (MP-mode) and 10^–5^ s (SP-mode). The full electrodeposition time (t_full_) was 30 s. The time of pause (t_off_) was equal to the pulse duration (t_on_) for all pulse modes. Standard silicon wafer with crystallography orientation of (100) were used as substrates for films deposition. Due to the insufficient conductivity of silicon, a gold layer with a thickness of 80 nm was preliminarily deposited on the substrate via ion-beam sputtering. The electrolyte composed of: NiSO_4_—210 g/l, NiCl_2_—20 g/l, H_3_BO_3_—30 g/l, MgSO_4_—60 g/l, FeSO_4_—15 g/l, saccharin—1 g/l with pH = 2.3–2.5 was used for the deposition of films with chemical composition Ni_80_Fe_20_. The electrodeposition process was carried our at current density of 25 mA/cm^2^ and electrolyte temperature of 30–35 °C. More information about electrodeposition parameters is given in Ref.^65^.

### AFM tip preparation

**Figure 6:**
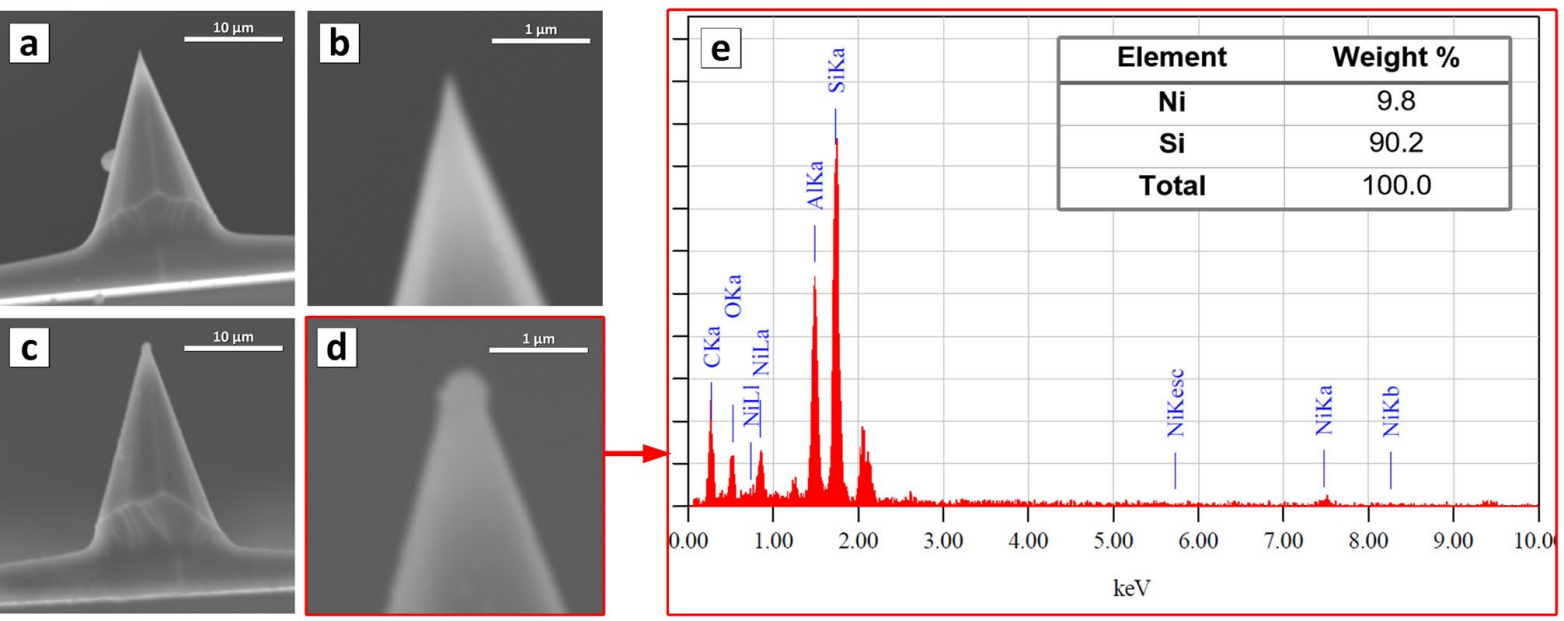
AFM probe for specific surface energy investigation: (**a**,**b**) Si tip witout coatings; (**c**,**d**) nickel coated (10 nm) tip; (**e**) chemical composition of the apex of the Ni coated tip.

A set of AFM probes was fabricated for the specific surface energy investigations and to simulate contact interaction. The standard Si probes NSC11 type with tip radius about 10 nm were fixed on the plate with the tip up. After that, the plate with the probes was coated with a thin Ni film (10 nm) by ion beam deposition. The Ni film covered both the plate and the probes. Figure 6 shows what the probe and tip looked like before (Fig. 6a,b) and after (Fig. 6c,d) Ni deposition. A drop of nickel accumulated on the tip of the probe. The Ni microdrop interacts with the investigated sample when using such modified probes. The radius of the modified probe tip that was used for present work is about 150 nm. The radius varied from 50 to 200 nm for other probes obtained with this one. The probe tip radius limits the minimal size of the investigated area but is not equal to it. The chemical composition analysis (Fig. 6e) showed that at the tip apex (which is shown in Fig. 6d) is almost 10 wt% nickel. As a result, the interaction between the Ni probe and Ni_80_Fe_20_ NGs on investigated sample is similar to the interaction of two Ni_80_Fe_20_ NGs. The state of the tip Ni coating was also monitored by SEM after using to exclude those experiments that caused the destruction of the coating.

### Methods of investigation

The surface microstructure was studied using the scanning electron microscope JCM-6000PLUS Neoscope (SEM) with Jeol microanalysis system and the atomic force microscope NT-206 (AFM). The atomic force microscope was designed in Nanoprocesses and Technologies Lab (A.V. Luikov Heat and Mass Transfer Institute of NAS of Belarus) for operation in a contact mode. The main objective of this device is to study the processes of friction and wear.

The root mean square roughness and average grain size of NiFe films were measured and calculated using at least three AFM images as in^66–68^. The calculation of average NGs size and other calculations were carried out taking the grains form as a sphere with an equivalent volume as in^67^. The Ni_80_Fe_20_ film thickness was investigated by stepwise abrasion with a diamond pyramidal tip using the nanoindenter TI 750 Ubi (Hysitron, USA). The chemical composition of the films was valuated by energy-dispersive X-ray microanalysis using a Bruker XFlash MIN SVE microanalyzer, working in conjunction with a Hitachi TM3030 SEM. The values of the normal specific surface energy (SSE) were determined with a widely known AFM technique called force spectroscopy^69,70^. The lateral SSE was studied using a unique technique based on recording the angle of the cantilever twist when scanning in contact with the surface, which was developed as part of this work (see paragraph 3.1). The scanning speed was 35 µm/s and the normal load was about 100 nN. Scanning speed and normal load are parameters that strongly influence the cantilever twist force during lateral movement. The nature of their influence on the research results requires careful Investigation for a deep understanding of the processes operating in the nanocontact, as well as for the development of appropriate corrections or the choice of optimal experimental conditions, which will be the goal of our further research.
